# Frailty severity is significantly associated with electrocardiographic QRS duration in chronic dialysis patients

**DOI:** 10.7717/peerj.1354

**Published:** 2015-10-22

**Authors:** Chia-Ter Chao, Jenq-Wen Huang

**Affiliations:** 1 Department of Medicine, National Taiwan University Hospital Jin-shan Branch, New Taipei City, Taiwan; 2 Graduate Institute of Toxicology, National Taiwan University, Taipei, Taiwan; 3 Department of Internal Medicine, National Taiwan University Hospital, Taipei, Taiwan

**Keywords:** Electrocardiography, Dialysis, Frailty, End-stage renal disease, QRS duration, Geriatrics, Sudden cardiac death, Arrhythmia, Simple FRAIL scale

## Abstract

End-stage renal disease (ESRD) patients are at increased risk of sudden cardiac death, the risk of which is presumably related to arrhythmia. Electrocardiographic (ECG) parameters have been found to correlate with arrhythmia and predict cardiovascular outcomes in ESRD patients. Frailty is also a common feature in this population. We investigate whether the severity of dialysis frailty is associated with ECG findings, including PR interval, QRS duration, and QTc interval. Presence and severity of frailty was ascertained using six different self-report questionnaires with proven construct validity. Correlation analysis between frailty severity and ECG was made, and those with significant association entered into multiple regression analysis for confirmation. Among a cohort of chronic hemodialysis patients, we found that frailty severity, assessed by the Edmonton frailty scale, is significantly associated with QRS duration (*r* = − 0.3, *p* < 0.05). Dialysis patients with QRS longer than 120 ms had significantly lower severity of frailty than those with QRS less than 120 ms (*p* = 0.01 for the Edmonton frailty scale and 0.05 for simple FRAIL scale). Regression analysis showed that frailty severity, assessed by the Edmonton frailty scale and simple FRAIL scale, was significantly associated with QRS duration independent of serum electrolyte levels. In conclusion, a significant relationship exists between the severity of frailty and QRS duration in ESRD patients. This might be an under-recognized link between frailty and its adverse cardiovascular impact in these patients.

## Introduction

Among chronic kidney disease (CKD) and end-stage renal disease (ESRD) patients, the combination of traditional risk factors (hypertension, diabetes mellitus (DM), and smoking) and non-classical features (inflammation, malnutrition, vascular calcification) contribute to the very high risk of cardiovascular events, especially sudden cardiac death (SCD) (Shlipak et al., 2005). Referring to death from presumable cardiac origin within one hour of symptom-onset, SCD claims nearly a quarter of all mortality cases in chronic dialysis patients, emerging as a serious public health concern (Passman & Herzog, 2011). Approximately half of these deaths are likely triggered by fatal arrhythmia (ventricular tachycardia/fibrillation), superimposed on susceptible substrates with myocardial ischemia or systolic dysfunction (Wang et al., 2010). Furthermore, electrocardiographic alterations, such as QTc interval alterations and repolarization dispersions, have been reported to be pathogenic in ventricular arrhythmia among chronic hemodialysis patients, serving as potential risk factors for SCD (Kalantzi et al., 2013).

Frailty, a phenotypic construct describing the low physiologic reserve status and stress vulnerability, is frequently associated with adverse outcomes in elderly patients with multimorbidity (Chao et al., 2015a). The emergence of frailty could involve aging-associated physical degeneration, as well as subclinical organ dysfunction resulting from intermittent insults. Increasing evidence suggests that frailty impairs the prognosis of not only patients of advanced age, but also those with chronic illnesses, especially CKD and ESRD, a pathologic status of accelerated ageing (Wilhelm-Leen et al., 2009). In addition, the presence of frailty is accompanied by a higher prevalence of coronary artery disease with more extensive vascular involvement, heart failure, and a higher risk of cardiovascular mortality (Gharacholou et al., 2012; Sanchis et al., 2014). Among a large cohort of chronic dialysis patients, Kutner and colleagues also showed that frailty is significantly associated with vascular morbidities (Kutner et al., 2014). It is interesting to observe that in the same study, cardiac dysrhythmia including atrial fibrillation, tachycardia, and cardiac arrest, exhibited significant association with frailty as well. The above findings raise the possibility that an association might exist between frailty and arrhythmia, leading to higher risk of cardiac death, including SCD, among CKD/ESRD patients. Indeed, frail patients are more likely to present untreated atrial fibrillation, potentially leading to adverse cardiac outcomes (Polidoro et al., 2013).

Since arrhythmic events among CKD/ESRD patients could be heralded by pre-existing electrocardiographic (ECG) abnormalities, studies have reported that common ECG parameters effectively predict cardiovascular mortality among patients with renal failure (Deo et al., 2015). ECG provides an aggregate overview of cardiac conduction and rhythm, and is convenient to obtain in clinical practice. We hypothesize that in ESRD patients, frailty might demonstrate significant association with their ECG parameters, thus potentially influencing the arrhythmogenic potential and the subsequent risk of cardiovascular mortality. We use a prospectively recruited chronic hemodialysis cohort to evaluate the existence of such association.

## Materials and Methods

### Participants and study design

We used a prospective observational cohort of chronic hemodialysis patients from National Taiwan University Hospital (NTUH) Jinshan branch for analysis (Chao et al., 2015a; Chao et al., 2015b). NTUH ethics committee approved this study (NO.201403006RINB) and all enrollees had verbal informed consent.

The status of frailty was ascertained using six different self-report questionnaires with Chinese-translation administered by trained nursing staffs, including Strawbridge questionnaire (frailty if >1 positive domain), Edmonton frailty scale (if score ≥8), Simple FRAIL scale (if score ≥3), Groningen frailty instrument (if score ≥4), G8 questionnaire (if score ≦14), and Tilburg frailty instrument (if score ≥5), as we previously reported (Chao et al., 2015a). All instruments demonstrated fair construct validity in multiple reports. The designation of instrument-specific frailty was according to the original criteria respectively, and a higher score of all but the G8 questionnaire indicates increasing severity of frailty.

### Outcomes

All participants received standard electrocardiographic examinations during their inter-dialytic period and after frailty status assessment. Resting electrocardiograms (ECGs) were recorded with a 12-channel Cardiofax Q electrocardiograph (Nihon Kohden, Tokyo, Japan) at a paper speed of 25 mm/sec as well as standardization (10 mm equals to 1 mV) with filter settings. Patients were mandated to have 5–10 min of rest before examination. Reproducibility of the ECGs was reassured with fixed marks for each lead and repeated examinations if deemed necessary by trained technicians. We collected electrocardiographic parameters including PR intervals, QRS durations, and corrected QT intervals (QTc) as provided by the interpretation programs of the ECG machine. ECG abnormalities, including inverted T waves, Q waves, atrial premature complexes (APCs), and ventricular premature complexes (VPCs) were also recorded for comparison.

### Statistical analysis

Continuous and categorical variables were expressed as mean ± standard deviation and the number with percentage, with comparisons between groups made by the independent *t*-test or Mann–Whitney *U*-test (the former) and the chi-square test (the latter) if appropriate. We assessed the correlation between clinical variables, including scores of the six frailty screening instruments, and the collected ECG parameters, using Pearson’s correlation coefficients. Variables exhibiting statistical significance correlation were selected for inclusion in the multivariate analysis. Multiple logistic regression analysis, incorporating clinical variables and all the frailty scores, were conducted to affirm the existence of associations between variables. In all statistical analyses, a two-sided *p* < 0.05 was considered significant.

## Results

### Clinical data of the participants

Among the forty-one chronic dialysis patients of advanced age (68.4 ± 10.8 years) and 44% male, more than four-fifth had pre-existing hypertension, followed by DM in nearly half, heart failure, and thyroid disease (Table 1). More than one-third patients had ESRD caused by diabetic nephropathy, followed by chronic glomerulonephritis. Laboratory data of this cohort were also shown in Table 1. Participants were normokalemia and normocalcemia (9.5 ± 1.6 meq/L and 9 ± 0.9 mg/dL), with low levels of inflammatory markers (C-reactive protein, 0.8 ± 1.3 mg/dL).

**Table 1:** Baseline characteristics of enrollees.

Clinial features	Results
*Demographic data*	
Age (years)	68.4 ± 10.8
Gender (male%)	18 (44)
Dialysis duration (years)	2.7 ± 2.5
*Comorbid conditions*	
Diabetes mellitus	19 (46)
Hypertension	35 (85)
Coronary artery disease	9 (22)
Heart failure	8 (20)
Malignancy	5 (12)
Thyroid diseases	5 (12)
*Liu’s dialysis comorbidity index*	1.6 ± 1.8
*Etiology of end-stage renal disease*	
Diabetic nephropathy	16 (39)
Chronic glomerulonephritis	3 (7)
Miscellaneous^a^	7 (17)
Unknown	15 (37)
*Laboratory data*	
Albumin (g/dL)	3.7 ± 0.3
Creatinine (mg/dL)	10.4 ± 2.5
Hemoglobin (mg/dL)	9.5 ± 1.6
Potassium (meq/L)	4.7 ± 0.9
Calcium (mg/dL)	9 ± 0.9
CRP (mg/dL)	0.8 ± 1.3
*Electrocardiographic parameters*	
PR interval (ms)	185.4 ± 28.1
QRS duration (ms)	98.2 ± 18.2
QTc interval (ms)	427.6 ± 27.3
Heart rate (per min)	85.3 ± 18
*Medication use*	
Beta-blockers	12 (29)

**Notes.**

Data were expressed as mean ± standard deviation for continuous variables, or number with percentages in parentheses for categorical variables.

a Including unrecovering acute kidney injury, systemic lupus erythematosus, chronic interstitial nephritis, allograft failure, obstructive uropathy.

Using the six screening instruments, we identified that 22% (simple FRAIL scale), 32% (Tilburg frailty indicator), 46% (Edmonton frailty scale), 56% (Groningen frailty indicator), 73% (Strawbridge questionnaire), and 83% (G8 questionnaire) patients were in a frail status. Frailty severity results assessed by five out of the six questionnaires (simple FRAIL scale, Tilburg frailty indicator, Edmonton frailty scale, Groningen frailty indicator, Strawbridge questionnaire) showed good correlation (correlation coefficient between 0.5 and 0.7), while results from G8 questionnaire showed poorer correlation with those of the others (Chao et al., 2015a).

Only one patient (2.4%) received ECG examination on the same day of the next session (40 h after the previous dialysis), while 97.6% patients received ECG at least one day after their previous sessions. ECG results showed a mean heart rate of 85.3 per minute among these patients. Four (9.8%) patients manifested atrial fibrillation on their ECGs, and PR intervals could not be determined in these patients. Mean PR intervals, excluding patients with atrial fibrillation, were 185.4 ± 28.1 ms, with about one-fourth (24.4%) of participants presenting PR prolongation (≥200 ms). QT prolongation (≥450 ms in male or ≥470 ms in female) among the entire cohort occurred in 4 patients (9.8%), while mean QTc intervals were 427.6 ± 27.3 ms. Five patients had prolonged QRS durations (≥120 ms). We found no significant differences among the obtained ECG parameters (PR interval, QRS duration, and QTc interval) between patients with and without heart failure. Beta-blocker users had significantly lower frailty severity assessed by simple FRAIL scale than non-users (the former vs. the latter, 0.9 vs. 1.8, *p* = 0.04).

Eleven patients (26.8%) had inverted T waves within at least one lead of their ECGs, while eight patients (19.5%), one patient (2.4%), and eight patients (19.5%) had significant Q waves, APCs, and VPCs within at least one lead of their ECGs. No significant difference was observed among the severity of frailty assessed by all the self-report frailty instruments between patients with and without inverted T waves. Similarly, no significant difference was observed among frailty severity between patients with and without significant Q waves, or with and without VPCs.

### Correlation between frailty severity and electrocardiographic parameters

**Figure 1:**
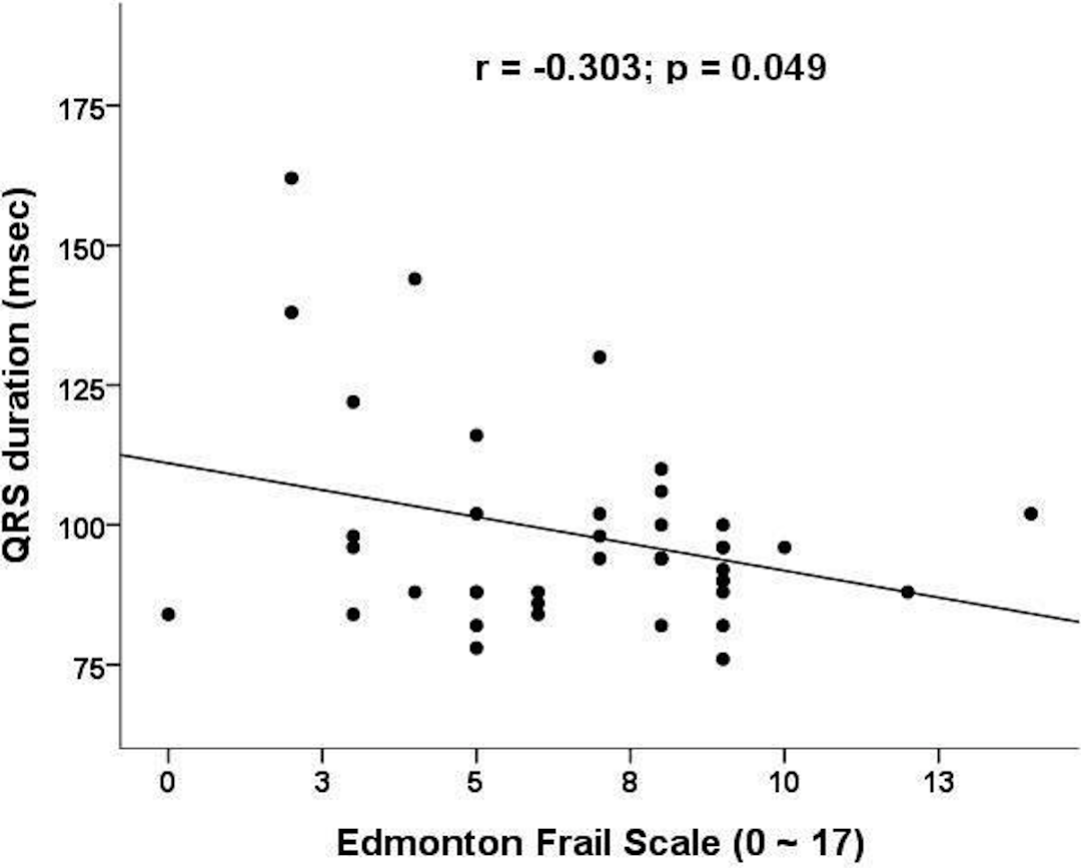
Correlation plot between frailty severity (assessed by Edmonton frailty scale) and QRS durations.

We next investigate the relationship between scores from the frailty-screening instruments and PR intervals/QRS duration/QTc intervals (Table 2). There was no significant correlation between the ECG parameters and laboratory data. We found that QRS duration correlated significantly with frailty severity, using scores from the Edmonton frailty scale (*p* = 0.049) (Fig. 1) and borderline significantly with the scores from Simple FRAIL scale (*p* = 0.08). Those with QRS longer than 120 ms have significantly lower frailty severity (by Edmonton frailty scale, longer vs. shorter, 3.6 vs. 7.1, *p* = 0.01; by simple FRAIL scale, 0.6 vs. 1.7, *p* = 0.05) and higher proportion of patients with frailty (by Edmonton frailty scale, longer vs. shorter, 0% vs. 53%, *p* < 0.01; by simple FRAIL scale, 0% vs. 25%, *p* = 0.01).

**Table 2:** Correlation between electrocardiographic parameters and clinical variables in the current cohort.

Coefficient (*p* value)	PR intervals	QRS duration	QTc interval
Albumin (g/dL)	0.05 (0.76)	0.26 (0.11)	0.13 (0.41)
Creatinine (mg/dL)	−0.02 (0.89)	0.2 (0.22)	0.02 (0.9)
Hemoglobin (mg/dL)	−0.09 (0.62)	0.28 (0.07)	0.15 (0.34)
Potassium (meq/L)	0.27 (0.11)	−0.07 (0.66)	−0.11 (0.51)
Calcium (mg/dL)	−0.07 (0.69)	0.23 (0.16)	0.05 (0.77)
CRP (mg/dL)	−0.25 (0.17)	−0.11 (0.54)	−0.17 (0.34)
Strawbridge questionnaire score	0.17 (0.33)	−0.05 (0.75)	−0.03 (0.84)
Edmonton Frailty Scale score	0.13 (0.46)	−0.3 (0.049)	−0.15 (0.36)
Simple FRAIL scale score	0.16 (0.34)	−0.28 (0.08)	−0.12 (0.47)
Groningen Frailty Indicator score	0.06 (0.73)	−0.11 (0.52)	−0.09 (0.59)
G8 questionnaire score	0.09 (0.58)	0.13 (0.43)	0.05 (0.75)
Tilburg Frailty Indicator score	−0.02 (0.89)	−0.07 (0.66)	−0.1 (0.53)

**Notes.**

CRP C-reative protein

### Regression analysis for relationship confirmation

We performed multiple regression analysis, with QRS duration as a dependent variable, to evaluate the relationship between QRS duration and the severity of frailty. Accounting for clinical parameters including heart failure, comorbidity index, and serum albumin/electrolyte levels, scores assessed by the Edmonton frailty scale showed significantly negative association with QRS duration (*β* coefficient = − 0.29, *t* = − 2.03, *p* = 0.048). Similarly, scores assessed by simple FRAIL scale showed significantly negative association with QRS duration as well (*β* coefficient = − 0.27, *t* = − 1.84, *p* = 0.05). A sensitivity analysis, incorporating interdialytic weight gain status (IDWG) (high, defined according to IDWG ≥5% of dry weight, or low) in the regression, did not alter our findings significantly (frailty severity by Edmonton frailty scale, *β* coefficient = − 0.58, *t* = − 3.42, *p* = 0.002).

## Discussion

In the current study, we found that self-report frailty is significantly associated with QRS duration in chronic hemodialysis patients, and the association is independent of serum electrolyte levels and heart failure status. This relationship potentially explains the higher risk of cardiovascular events among patients with frailty, and provides an opportunity to detect the adverse impact of frailty at an earlier stage.

Frailty significantly raises the risk of cardiovascular death among those with cardiac morbidities (Ekerstad et al., 2011). However, the mediator of this frailty-introduced cardiovascular disadvantage is rarely elucidated before, and never in ESRD population. Past reports discovered that QRS prolongation is predictive of increased mortality among patients with hypertension, coronary artery disease, and heart failure (Elhendy et al., 2005). Electrophysiological studies on myocardium showed that intraventricular conduction delay correlates significantly with patchy and diffuse myocardial fibrosis, resulting in QRS lengthening (Kawara et al., 2001). Consequently, the significant association between frailty severity and QRS duration could signify that, through its relationship with QRS duration and potentially arrhythmogenic potential, frailty contributes to the observed cardiovascular risk in ESRD patients.

Hemodialysis poses a significant challenge to ESRD patients, affecting their hemodynamic stability through rapid volume removal. However, apart from its hemodynamic effect, dialysis might induce alterations in ECG findings. Several reports suggest that hemodialysis is associated with increases in T wave, QRS amplitude, and prolongation of QTc interval and QRS duration (Covic et al., 2002; Madias, 2006). The main reason for these ECG changes has been proposed to be related to fluid status instead of changes in electrophysiology (Madias, 2006). Post-dialysis ECG parameters (duration/amplitude) are often accentuated due to amelioration of edema from ultrafiltration. Indeed, malnutrition is frequently accompanied by frailty, and ESRD patients with overhydration have significantly higher proportion of malnutrition (Eyigor et al., 2015). Malnutrition-inflammation syndrome in chronic dialysis patients also correlates significantly with excess extracellular fluid and an edematous status (Guo et al., 2013). Consequently, the inverse relationship between QRS duration and frailty severity found in this study might suggest that ESRD patients with more severe frailty exhibit higher levels of fluid accumulation than those with less severe frailty, leading to a shorter QRS duration on their ECG. Since all ECG in this study were obtained during intra-dialytic period, the parameters would not be affected by the dialysis procedure *per se*. This association is independent of electrolyte (potassium and calcium) and heart failure status, also lending support to this frailty-malnutrition-edema theory in chronic dialysis patients.

The frequency of hemodialysis might potentially influence the risk of arrhythmia. Studies reported that complex arrhythmic episodes, including premature ventricular contractions, tend to occur within the first 6–12 h after each session of dialysis (Santoro et al., 2008). It is then plausible that patients receiving lower frequency of dialysis per week are at less risk of dialysis-related arrhythmia than those receiving higher frequency of treatment, and differences in their inter-dialytic ECG parameters might exist. In our cohort, only 2 patients (4.9%) received twice weekly dialysis, while others (95.1%) received thrice weekly dialysis. The two patients under twice weekly dialysis did not exhibit PR prolongation, and their QTc intervals and QRS durations were normal. Excluding these two patients from analysis, we similarly found that dialysis patients with QRS prolongation has significantly lower frailty severity (using the Edmonton scale, longer vs. shorter, 3.6 vs. 7.0, *p* = 0.01; using simple FRAIL scale, 0.6 vs. 1.7, *p* = 0.05). These findings suggest that the frequency of dialysis session did not affect our results significantly.

Atrial fibrillation is also common in chronic dialysis patients, and QRS duration could be an important outcome-predictor in those with atrial fibrillation (Lin, Liu & Chu, 2009; Chao et al., 2012). Similarly, chronic dialysis patients are at risk of developing left ventricular hypertrophy (LVH), and existing literature identified a potential association between LVH and QRS prolongation, especially in hypertensive patients (Budhwani, Patel & Dwyer, 2005). However, incorporating these variables (atrial fibrillation, LVH or not) in our regression did not affect the relationship we identified. This suggests that these two factors play a minor role in the association between the frailty severity and QRS durations.

Our study had its limitations. The case number in this cohort is modest, attenuating the statistical power for accurate analysis; the significance of our finding could increase the credibility of this study. The utilization of different self-report frailty instruments for assessing frailty severity and repetitive ECG examination for reproducibility also strengthens our results. In addition, the absence of echocardiographic or cardiac magnetic resonance findings for myocardial status ascertainment reduces the interpretability of our findings, and administration of self-report questionnaires might suffer from recall bias. Further large-scale study is needed to confirm and also extend the applicability of our results.

## Supplemental Information

10.7717/peerj.1354/supp-1 Supplemental Information 1 Raw data
